# Patterns of interval correlations in neural oscillators with adaptation

**DOI:** 10.3389/fncom.2013.00164

**Published:** 2013-11-29

**Authors:** Tilo Schwalger, Benjamin Lindner

**Affiliations:** ^1^Bernstein Center for Computational NeuroscienceBerlin, Germany; ^2^Department of Physics, Humboldt Universität zu BerlinBerlin, Germany

**Keywords:** spike-frequency adaptation, non-renewal process, serial correlation coefficient, phase-response curve, integrate-and-fire model, long-term variability

## Abstract

Neural firing is often subject to negative feedback by adaptation currents. These currents can induce strong correlations among the time intervals between spikes. Here we study analytically the interval correlations of a broad class of noisy neural oscillators with spike-triggered adaptation of arbitrary strength and time scale. Our weak-noise theory provides a general relation between the correlations and the phase-response curve (PRC) of the oscillator, proves anti-correlations between neighboring intervals for adapting neurons with type I PRC and identifies a single order parameter that determines the qualitative pattern of correlations. Monotonically decaying or oscillating correlation structures can be related to qualitatively different voltage traces after spiking, which can be explained by the phase plane geometry. At high firing rates, the long-term variability of the spike train associated with the cumulative interval correlations becomes small, independent of model details. Our results are verified by comparison with stochastic simulations of the exponential, leaky, and generalized integrate-and-fire models with adaptation.

## 1. Introduction

The nerve cells of the brain are complex physical systems. They generate action potentials (spikes) by a nonlinear, adaptive, and noisy mechanism. In order to understand signal processing in single neurons, it is vital to analyze the sequence of the interspike intervals (ISIs) between adjacent action potentials. There is experimental evidence accumulating that the spiking in many cases is *not* a renewal process, i.e., a spike train with mutually independent ISIs, but that intervals are typically correlated over a few lags (Lowen and Teich, 1992; Ratnam and Nelson, 2000; Neiman and Russell, 2001; Nawrot et al., 2007; Engel et al., 2008) [further reports are reviewed in (Farkhooi et al., 2009; Avila-Akerberg and Chacron, 2011)]. These correlations are a basic statistics of any spike train with important implications for information transmission and signal detection in neural systems (Ratnam and Nelson, 2000; Chacron et al., 2001, 2004; Avila-Akerberg and Chacron, 2011) and man-made signal detectors (Nikitin et al., 2012). They are often characterized by the serial correlation coefficient (SCC)

(1) $$\rho_{k}=\frac{\langle \left(T_{i}-\langle T_{i}\rangle \right)\left(T_{i+k}-\langle T_{i+k}\rangle \right)\rangle }{\langle {\left(T_{i}-\langle T_{i}\rangle \right)}^{2}\rangle },$$

where *T*_*i*_ and *T*_*i* + *k*_ are two ISIs lagged by an integer *k* and 〈·〉 denotes ensemble averaging. ISI correlations can be induced via correlated input to the neural dynamics, e.g. in the form of external colored noise (Middleton et al., 2003; Lindner, 2004), intrinsic noise from ion channels with slow kinetics (Fisch et al., 2012), or stochastic narrow-band input (Neiman and Russell, 2001, 2005; Bauermeister et al., 2013).

Another ubiquitous mechanism for ISI correlations are slow feedback processes mediating spike-frequency adaptation (Chacron et al., 2000; Liu and Wang, 2001; Benda et al., 2005)—a phenomenon describing the reduced neuronal response to slowly changing stimuli (Benda and Herz, 2003; Gabbiani and Krapp, 2006). In the stationary state, these adaptation mechanisms are typically associated with short-range correlations with a negative SCC at lag *k* = 1 and a reduced Fano factor as demonstrated by several numerical (Geisler and Goldberg, 1966; Wang, 1998; Liu and Wang, 2001; Benda et al., 2010) and analytical studies (Schwalger et al., 2010; Schwalger and Lindner, 2010; Farkhooi et al., 2011; Urdapilleta, 2011). The correlation structure of adapting neurons can show qualitatively different patterns, ranging from monotonically decaying correlations to damped oscillations when plotted as a function of the lag (Ratnam and Nelson, 2000). Because ISI correlations shape spectral measures (Chacron et al., 2004), they bear implications for neural computation in general. However, a simple theory that predicts and explains possible correlation patterns is still lacking.

In this article, we present a relation between the ISI correlation coefficient ρ_*k*_ and a basic characteristics of nonlinear neural dynamics, the *phase-response curve* (PRC). The PRC quantifies the advance (or delay) of the next spike caused by a small depolarizing current applied at a certain time after the last spike (Ermentrout, 1996). For neurons which integrate up their input (integrator neurons), the PRC is positive at all times (type I PRC) whereas neurons, which show subthreshold resonances (resonator neurons), possess a PRC that is partly negative (type II PRC) (Ermentrout, 1996; Izhikevich, 2005; Ermentrout and Terman, 2010). Below we show that resonator neurons possess a richer repertoire of correlation patterns than integrator neurons do.

## 2. Results

### 2.1. Model

Spike frequency adaptation can be modeled by Hodgkin–Huxley type neurons with a depolarization-activated adaptation current (Wang, 1998; Ermentrout et al., 2001; Benda and Herz, 2003). However, the spiking of such conductance-based models can in many instances be approximated by simpler multi-dimensional integrate-fire (IF) models that are equipped with a spike-triggered adaptation current (Treves, 1993; Izhikevich, 2003; Brette and Gerstner, 2005); adapting IF models perform excellently in predicting spike times of real cells under noisy stimulation (Gerstner and Naud, 2009). Here, we consider a stochastic nonlinear multi-dimensional IF model for the membrane potential *v*, *N* auxiliary variables *w*_*j*_ (*j* = 1, …, *N*) and a spike-triggered adaption current *a*(*t*):

(2a) $$\;\dot{v}=f_{0}\text{​}\left(v,\;w\right)+\mu -a+\xi (t),$$

(2b) $$\;{\dot{w}}_{j}=f_{j}(v,\;w),$$

(2c) $$\;\tau_{a}\dot{a}=-a+\tau_{a}\Delta \sum_{i}\delta \left(t-t_{i}\right).$$

The membrane potential *v*(*t*) is subject to weak Gaussian noise ξ(*t*) with 〈ξ(*t*)ξ(*t*′)〉 = 2*D*δ(*t* − *t*′) and noise intensity *D*. The dynamics is complemented by a spike-and-reset mechanism: whenever *v*_(T)_ reaches a threshold υ(*t*), a spike is registered at time *t*_*i*_ = *t* and *v*(*t*) and **w**(*t*) = [*w*_1_(*t*), …, *w*_*N*_(*t*)]^T^ are reset to *v*(*t*^+^_*i*_) = 0 and **w**(*t*^+^_*i*_) = **w**_*r*_ (where *t*^+^_*i*_ denotes the right-sided limit *t* → *t*_*i*_ + 0). At the same time, *a*(*t*) suffers a jump by Δ ≥ 0 as seen from Equation (2c), which resembles high-threshold adaptation currents (Wang, 1998; Liu and Wang, 2001). The constant input current μ is assumed to be sufficiently large to ensure ongoing spiking even in the absence of noise. Note that the model is non-dimensionalized by measuring time in units of the membrane time constant τm~ 10 ms and voltage in units of the distance between reset and spike-initiating potential (a typical value is 15 mV). In particular, the adaptation time constant τ_*a*_ is measured relative to τm and the unit of the firing rate is τ^−1^_*m*_ ~ 100 Hz.

**Figure 1:**
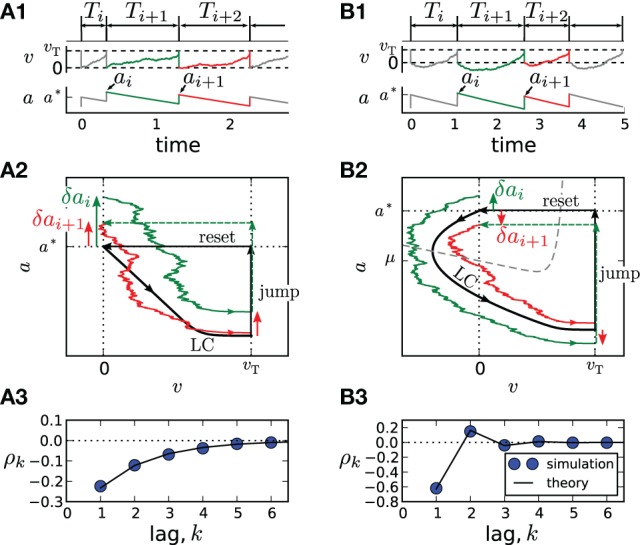
**Correlation patterns in the adaptive exponential IF model with τ_*a*_ = 10,** γ **= 1, Δ_*T*_ = 0.1,** υ**_**T**_ = 2, *D* = 0.1**. Adaptation is weak (Δ = 1, μ = 15) in **(A)** and strong (Δ = 10, μ = 80) in **(B)**. Membrane voltage *v*(*t*) and adaptation variable *a*(*t*) with ISI sequences {*T*_*i*_} and peak adaptation values {*a*_*i*_} are shown in **(A1,B1)**; time is in units of the membrane time constant τm. Colored pieces of trajectories in the phase plane (v,a) in **(A2,B2)** correspond to the respective colors in **(A1,B1)**. The deterministic limit cycle (LC), determined by the initial (post-spike) values *v* = 0, *a* = a^*^, is indicated by a thick black line. For weak adaptation **(A2)** a short ISI *T*_*i*_ causes positive deviations δ*a*_*i*_ = *a*_*i*_ − a^*^ and δ*a*_*i* + 1_ = *a*_*i* + 1_ − a^*^ of peak values leading to long ISIs *T*_*i* + 1_ and *T*_*i* + 2_ and, hence, to a negative ISI correlation at all lags **(A3)**. Because of the qualitatively different limit cycle for strong adaptation **(B2)**, deviations δ*a*_*i*_ and δ*a*_*i* + 1_ differ in sign, yielding an oscillatory correlation pattern **(B3)**.

An important special case, the adaptive exponential integrate-and-fire model (Brette and Gerstner, 2005) with purely spike-triggered adaptation and a white noise current with constant mean is illustrated in Figure 1. It assumes an exponential nonlinearity *f*_0_(*v*) = −γ*v* + γΔ_*T*_ exp[(*v* − 1)/Δ_*T*_] (Fourcaud-Trocmé et al., 2003; Badel et al., 2008) and corresponds to *N* = 0. Time courses of *v*(*t*) and *a*(*t*) are shown in Figures 1A1,B1 for two distinct correlation patterns possible in this model. The ISIs *T*_*i*_ = *t*_*i*_ − *t*_*i* − 1_ are obtained as differences between subsequent spiking times *t*_*i*_. The sequence *T*_*i*_, *T*_*i* + 1_, *T*_*i* + 2_ displays patterns of *short-long-long* (Figure 1A1) and *short-long-short* (Figure 1B1), corresponding to a negative SCC, which decays monotonically with the lag *k* (Figure 1A3) or to an SCC oscillating with *k* (Figure 1B3). In the following, we develop a theory to analyze these and other correlation patterns possible in multi-dimensional adapting IF models.

### 2.2. General theory

In our model Equation (2), *a*(*t*) is the only variable that keeps a memory of the previous spike times thereby inducing correlations between ISIs. Over one ISI the time course of adaptation is an exponential decay, relating two adjacent peak values *a*_*i*_ = *a*(*t*^+^_*i*_) and *a*_*i* + 1_ = *a*(*t*^+^_*i* + 1_) by

(3) $$a_{i+1}=a_{i}e^{-T_{i+1}/\tau_{a}}+\Delta$$

(Figures A1,B1). We assume that in the deterministic case (*D* = 0) our model has a finite period T^*^ (i.e., the model operates in the tonically firing regime) and, hence, for *D* = 0 the map (3) has a stable fixed point

(4) $$a^{*}=\Delta /\left[1-\exp\left(-T^{*}/\tau_{a}\right)\right].$$

The asymptotic deterministic dynamics can be interpreted as a limit-cycle like motion in the phase space from the reset point to the threshold and back by the instantaneous reset [cf. Figures 1A2,B2].

Weak noise will cause small deviations in the period δ*T*_*i*_ = *T*_*i*_ − T^*^≈ *T*_*i*_ − 〈*T*_*i*_〉 that are mutually correlated with coefficient ρ_*k*_ = 〈δ*T*_*i*_δ*T*_*i* + *k*_〉/〈δ*T*^2^_*i*_〉. The peak adaptation values, however, also fluctuate, δ*a*_*i*_ = *a*_*i*_ − a^*^, and both deviations are related by linearizing Equation (3):

(5) $$\delta T_{i+1}=\frac{\tau_{a}}{a^{*}}\left(\delta a_{i}-e^{T^{*}/\tau_{a}}\delta a_{i+1}\right).$$

A second relation between the small deviations can be gained by considering how a small perturbation in the voltage dynamics affects the length of the period. This effect is captured by the infinitesimal phase response curve (PRC), *Z*(*t*), *t* ∈ (0, T^*^) (Izhikevich, 2005; Ermentrout and Terman, 2010) (see Section 4 for the precise definition). During the interval *T*_*i* + 1_, the voltage dynamics in Equation (2a) can be written as $\dot{v}=f_{0}(v,\;w)+\mu -(a^{*}+\delta a_{i})e^{-(t-t_{i})/\tau_{a}}+\xi (t)$. Compared to the deterministic limit cycle, the dynamics is perturbed by the weak noise and the small deviation in the adaptation δ*a*_*i*_*e*^−(*t* − *t*_*i*_)/τ_*a*_^ yielding in linear response

(6) $$\delta T_{i+1}=\int_{0}^{T^{*}}\text{d}t\;Z(t)\left(\delta a_{i}e^{-\frac{t}{\tau_{a}}}-\xi (t_{i}+t)\right).$$

Combining Equations (5), (6) we obtain the stochastic map

(7) $$\delta a_{i+1}=\alpha \vartheta \delta a_{i}+\Xi_{i},$$

where $\Xi_{i}=\frac{\alpha a^{*}}{\tau_{a}}\int_{0}^{\infty }\text{d}t\;Z(t)\xi (t_{i}+t)\;$ are uncorrelated Gaussian random numbers and

(8) $$\alpha =e^{-T^{*}/\tau_{a}},\;\vartheta =1-\frac{a^{*}}{\tau_{a}}\int_{0}^{T^{*}}\text{d}t\;Z(t)e^{-\frac{t}{\tau_{a}}}.$$

Note that local stability of the fixed point a^*^ requires that |αϑ| < 1. The covariance *c*_*k*_ = 〈δ*a*_*i*_δ*a*_*i* + *k*_〉 of the auto-regressive process Equation (7) can be calculated by elementary means and using Equation (5) we obtain for *k* ≥ 1:

(9) $$\rho_{k}=-A(1-\vartheta ){\left(\alpha \vartheta \right)}^{k-1},\;A=\frac{\alpha (1-\alpha^{2}\vartheta )}{1+\alpha^{2}-2\alpha^{2}\vartheta }.$$

In order to compute α and ϑ via Equation (8), we have to calculate T^*^ and *Z*(*t*) (a^*^ then follows from Equation (4)), which can be done for simple systems analytically.

Our main result, Equations (8), (9), allows to draw a number of general conclusions. It shows that the SCC is always a geometric sequence with respect to the lag *k* that can generate qualitatively different correlation patterns depending on the value of ϑ and thus on PRC and adaptation current. Because |αϑ| < 1 and 0 < α < 1, the prefactor *A* in Equation (9) is always positive. Consequently, ρ_1_ is negative for ϑ < 1 and positive for ϑ > 1. Looking at Equation (8), we find that a positive PRC inevitably yields ϑ < 1. This implies that adapting neurons with type I PRC possess negative correlations between adjacent ISIs. Intuitively, a short ISI causes in the following on average a higher inhibitory adaptation during the subsequent ISI. Such an inhibitory current always enlarges the ISI in type I neurons—hence, a short ISI is followed by a long ISI.

The sign of higher lags is determined by the base of the power: for ϑ > 0 correlations decay monotonically, whereas for ϑ < 0 the SCC oscillates. Two special cases are ϑ = 0 with a negative correlation at lag 1 and vanishing correlations at all higher lags and ϑ = 1 where all correlations vanish. Overall, we find five basic patterns corresponding to the cases −α^−1^ < ϑ < 0, ϑ = 0, 0 < ϑ < 1, ϑ = 1 and 1 < ϑ < α^−1^. These basic patterns cover all interval correlations discussed in previous theoretical studies (Schwalger and Lindner, 2010; Urdapilleta, 2011). Our geometric formula generalizes the theory for the perfect IF model with adaptation (Schwalger et al., 2010) to more realistic, nonlinear multi-dimensional IF models with adaptation.

The cumulative effect of the correlations can be described by the sum over all ρ_*k*_, which determines the long-time limit of the Fano factor and the low-frequency limit of the spike train power spectrum (for a definition of these quantities, see Section 4.2). Evaluating the geometric series yields

(10) $$\sum_{k=1}^{\infty }\rho_{k}=-\frac{A\left(1-\vartheta \right)}{1-\alpha \vartheta }.$$

This shows that adaptation in neurons with type I resetting (ϑ < 1) leads to a negative summed correlation and hence a reduced long-term variability. Furthermore, at high firing rates achieved by a strong input current μ, the sum in Equation (10) can be approximated by

(11) $$\sum_{k=1}^{\infty }\rho_{k}≃-\frac{1}{2}+\frac{1/2}{{\left(1+\Delta \tau_{a}/v_{\text{T}}\right)}^{2}},\;T^{*}\ll \tau_{a}.$$

In particular, for strong adaptation (Δτ_*a*_ ≫ *v*_T_) the sum is only slightly larger than −1/2. Note that by virtue of the fundamental relation $\lim_{t\to \infty }F(t)=C_{\text{V}}^{2}\left(1+2\sum_{k=1}^{\infty }\rho_{k}\right)$ (Cox and Lewis, 1966) (see Section 4.2), the smallest possible value for the sum is −1/2 in order to ensure the non-negativity of the Fano factor *F*(*t*). At this minimal value the long-term variability as expressed by the Fano factor vanishes even for a non-vanishing ISI variability as quantified by the coefficient of variation *C*_V_. The latter quantity can also be estimated using the weak-noise theory: From Equation (7) one can calculate the variance of *a*_*i*_ and using Equation (5) an approximation for *C*^2^_V_ ≈ 〈δ*T*^2^_*i*_〉/T^*^2^^ can be obtained as follows:

(12) $$C_{\text{V}}^{2}=2D\frac{1+\alpha^{2}-2\alpha^{2}\vartheta }{[1-{\left(\alpha \vartheta \right)}^{2}]T^{*}{}^{2}}\int_{0}^{T^{*}}\text{d}t\;{\left[Z(t)\right]}^{2}.$$

### 2.3. One-dimensional IF models with adaptation

In the simplest case (*N* = 0, *f*_0_(*v*, **w**) = *f*(*v*)) the PRC reads

(13) $$Z(t)=Z(T^{*})\;\exp[\int_{t}^{T^{*}}\text{d}t^{\prime }\;f^{\prime }(v_{0}(t^{\prime }))],$$

where *v*_0_(*t*) is the limit cycle solution and *Z*(T^*^) = [*f*(*v*_*T*_) + μ − a^*^ + Δ]^−1^ is the inverse of the velocity ${\dot{v}}_{0}(T^{*})$ at the threshold, which is always positive. Thus, the PRC is positive for all *t* ∈ (0, T^*^), i.e., one-dimensional IF models show type I behavior. From our general considerations, this implies a negative SCC at lag *k* = 1. The sign of the correlations at higher lags can be inferred from the sign of ϑ, for which one can show (Section 4) that

(14) $$\vartheta =\left(f(0)+\mu -a^{*}\right)Z(0).$$

Because *Z*(0) > 0, the sign of ϑ is determined by the sign of *f*(0) + μ − a^*^. For weak adaptation such that a^*^ < *f*(0) + μ (achieved by a sufficiently small value of Δ or τ_*a*_, Figure 1A), we will have ϑ > 0 and a negative correlation at all lags (Figure 1A3). In this case, a short ISI occurring by fluctuation will cause a positive deviation δ*a*_*i*_ (Figure 1A2, green arrow). Geometrically, it is plausible that such a positive deviation causes a likewise positive deviation δ*a*_*i* + 1_ in the subsequent cycle (Figure 1A2, red arrow). Because a positive deviation is associated with a long ISI, the initial short ISI is on average followed by longer ISIs.

In marked contrast, for strong adaptation such that a^*^ > *f*(0) + μ (achieved by a sufficiently large value of Δ or τ_*a*_), ϑ becomes negative and hence the SCC's sign alternates with the lag. This alternation of the sign can be understood by means of the phase plane. Let us again consider a positive deviation δ*a*_*i*_ due to a short preceding ISI (Figure 1B2, green arrow). Because ${\dot{v}}_{0}(0)=f(0)+\mu -a^{*}<0$, the neuron is reset above the *v*-nullcline and hence hyperpolarizes at the beginning of the interval, i.e., the trajectory makes a detour into the region of negative voltage (corresponding to a “broad reset” in Naud et al. (2008)). A positive deviation δ*a*_*i*_ leads to a larger detour (green trajectory) causing a sign inversion and hence a negative deviation δ*a*_*i* + 1_ (Figure 1B2, red arrow). Because a positive (negative) deviation corresponds on average to a long (short) ISI, the alternation of δ*a*_*i*_ also entails an alternation of the ISI correlations. Thus, the distinction between monotonic and alternating patterns relates to a qualitative distinction of the voltage trace after resetting [cf. “sharp” vs. “broad” resets in Naud et al. (2008)].

**Figure 2:**
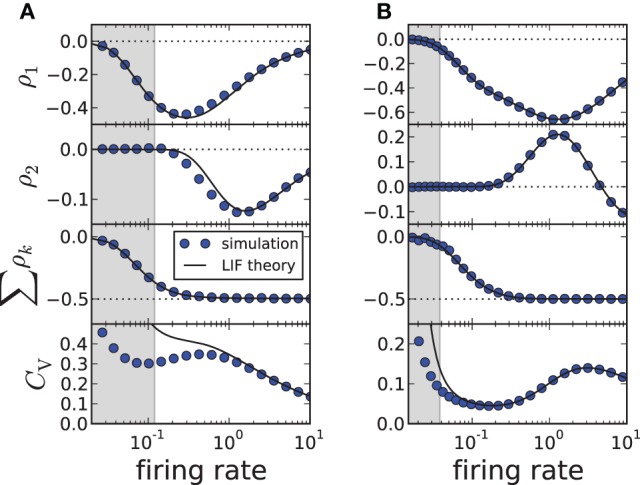
ISI correlations and coefficient of variation (CV) of the adapting LIF model vs. firing rate 1/〈*T*_*i*_〉≈ 1/T^*^, where the rate is varied by increasing μ. The gray-shaded area corresponds to the fluctuation-driven regime (μ < γ*v*_*T*_), where the assumptions of the theory do not hold. The panels display (from top to bottom) ρ_1_, ρ_2_, the sum $\sum_{k=1}^{m}\rho_{k}$ and the CV for simulation (circles, *m* = 100) and theory (solid lines, *m* → ∞). **(A)** Moderate adaptation: Δ = 1, **(B)** strong adaptation: Δ = 10. Both: γ = 1, τ_*a*_ = 10, *D* = 0.1, *v*_*T*_ = 1. Note that the firing rate is given in units of the inverse membrane time constant τ^−1^_*m*_.

As demonstrated in Figures 1A3,B3, our theory works well for the adapting exponential integrate-and-fire model. We next demonstrate the validity of our approach over a broad range of firing rates (Figure 2) for another important 1D model, the adapting leaky integrate-and-fire model (Treves, 1993) for which *f*(*v*) = −γ*v* and

(15) $$Z(t)=\exp\left[\gamma (t-T^{*})\right]/\left(\mu -\gamma v_{\text{T}}-a^{*}+\Delta \right)$$

(here T^*^ has still to be determined from a transcendental equation). Changing the firing rate by varying the input current μ, we find a good agreement for the first two correlation coefficients and the sum of all ρ_*k*_; the approximation of the CV shows deviations from simulation results when the input current μ becomes small (approaching the fluctuation-driven regime). In accordance with previous findings (Wang, 1998; Liu and Wang, 2001; Benda et al., 2010; Nesse et al., 2010; Schwalger et al., 2010; Schwalger and Lindner, 2010; Urdapilleta, 2011), the first correlation coefficient ρ_1_ displays a minimum corresponding to strong anti-correlations between adjacent intervals. The correlations at lag 2 can be positive for a finite range of firing rates if the adaptation strength is sufficiently large (Figure 2B), whereas for moderate adaptation we find a negative ρ_2_ at all firing rates (Figure 2A). In both cases, however, the sum of SCCs approaches a value close to −1/2 for high firing rates as predicted by Equation (11) (Figure 2, bottom). This is strikingly similar to experimental data from weakly electric fish, in which some electro-receptors display a monotonically decaying SCC and some show an oscillatory SCC (Ratnam and Nelson, 2000) but all cells exhibit a sum close to −1/2 (Ratnam and Goense, 2004). Finally, Figure 2 reveals a local maximum of the CV for some suprathreshold current μ—an effect that has been described by Nesse et al. (2008).

### 2.4. Generalized integrate-and-fire model with adaptation

Different correlation patterns become possible if we consider a type II PRC, which is by definition partly negative and can lead to a negative value of the integral in Equation (8), and hence to ϑ ≥ 1. This corresponds to a non-negative SCC at lag 1, which is infeasible in the one-dimensional case. To test the prediction ρ_1_ ≥ 0, we study the generalized integrate-and-fire (GIF) model (Brunel et al., 2003) with spike-triggered adaptation. This model is defined by *f*_0_(*v*, *w*) = −γ*v* − β*w* and *f*_1_(*v*, *w*) = (*v* − *w*)/τ_*w*_. Using the method described in Section 4, the PRC is obtained as

(16) $$Z(t)=\frac{e^{\frac{\nu }{2}(t-T^{*})}\left[\cos(\Omega (t-T^{*}))-\frac{1-\tau_{w}\gamma }{2\tau_{w}\Omega }\sin\left(\Omega (t-T^{*})\right)\right]}{\mu -\gamma v_{\text{T}}-\beta w_{0}(T^{*})-a^{*}+\Delta }$$

where $\nu =\gamma +1/\tau_{w},\;\Omega =\sqrt{\frac{\beta +\gamma }{\tau_{w}}-\frac{\nu^{2}}{4}}$ and *w*_0_(*t*) is one component of the deterministic limit-cycle solution [*v*_0_(*t*), *w*_0_(*t*), *a*_0_(*t*)] that we calculated numerically.

**Figure 3:**
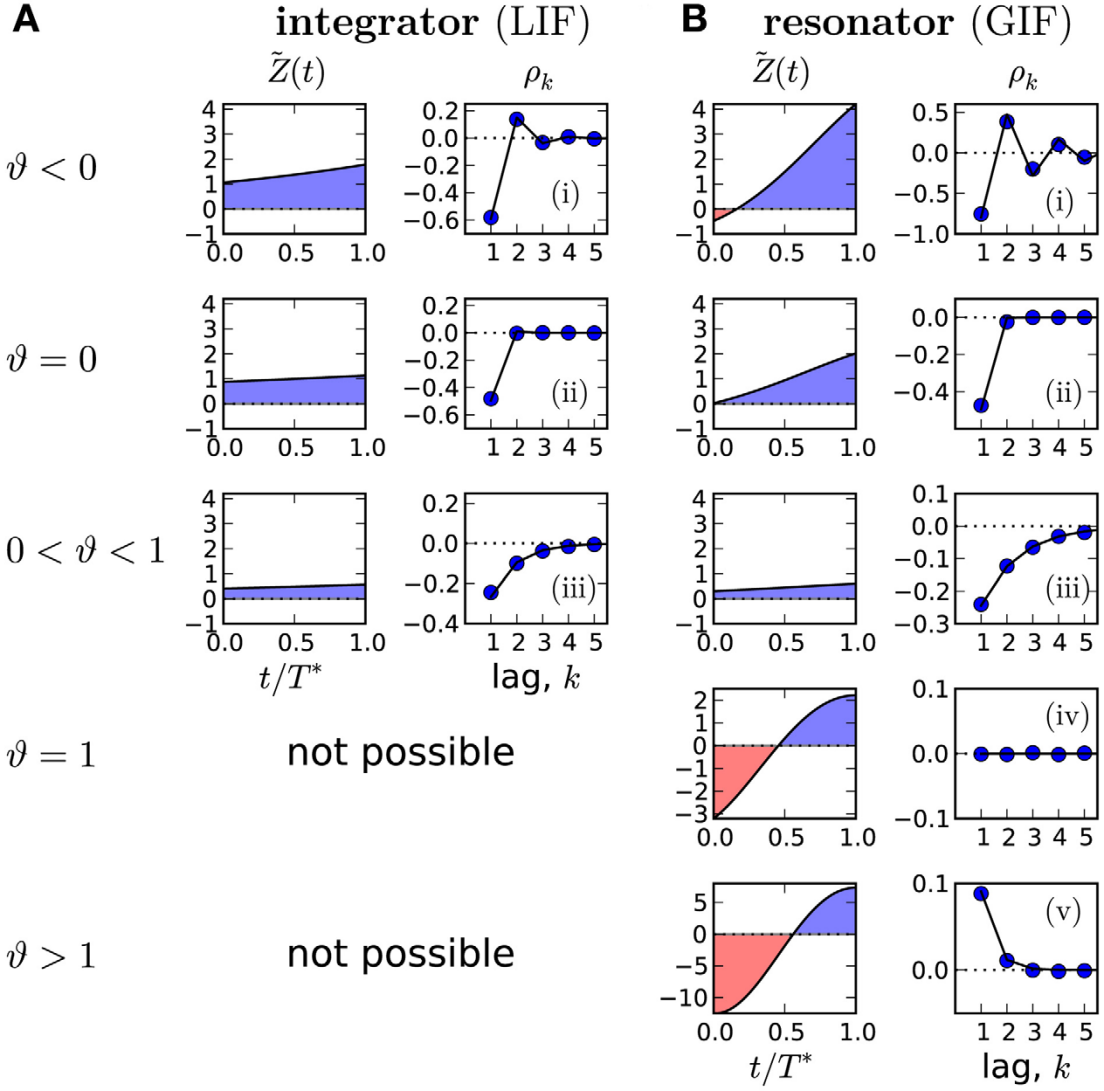
**Possible correlation patterns and corresponding PRCs (solid lines: theory, symbols: simulations of Equation** (2)**)**. For the adapting LIF model **(A)**, ϑ < 1 and only three qualitative different cases are possible. The adapting GIF model **(B)** exhibits the full repertoire of correlation patterns because the PRC can be partly negative and ϑ can attain values from its entire physically meaningful interval [−1/α, 1/α]. The value of ϑ and hence the type of correlation pattern is set by the integral over the weighted PRC $\tilde{Z}(t)=Z(t)e^{-\frac{t}{\tau_{a}}}\frac{a^{*}T^{*}}{\tau_{a}}$, shown in left panels. LIF parameters: *D* = 0.1, τ_*a*_ = 2, **(i)** μ = 20, Δ = 10, **(ii)** μ = 20, Δ = 4.47, (iii) μ = 5, Δ = 1. GIF parameters: **(i)** μ = 10, β = 3, τ_*a*_ = 10. **(ii)** μ = 11.75, β = 3, τ_*a*_ = 10. **(iii)** μ = 20, β = 1.5, τ_*a*_ = 10. **(iv)** μ = 2.12, β = 1.5, τ_*a*_ = 1, Δ = 10. **(v)** μ = 1.5, β = 1.5, τ_*a*_ = 1, Δ = 9 *D* = 10^−5^. Unless stated otherwise, γ = 1, Δ = 1, τ_*w*_ = 1.5, *D* = 10^−4^, *w*_*r*_ = 0.

In Figure 3B we demonstrate that all possible correlation patterns can be realized in the GIF model and that the predicted SCCs agree quantitatively well in theory and model simulations (for comparison, see the SCC for the LIF in Figure 3A). To each distinct pattern belongs a range of ϑ (Figure 3, left), determined by the area under the weighted PRC $\tilde{Z}(t)=\frac{a^{*}}{\tau_{a}}e^{-\frac{t}{\tau_{a}}}Z(t)$. The function $\tilde{Z}(t)$ (left column in Figures 3A,B) illustrates, why an adapting GIF neuron can show vanishing (Figure 3Biv) or even *purely positive* ISI correlations (Figure 3Bv). In case of type II resetting, inhibitory input can *shorten* the ISI because of the negative part in the PRC; here inhibition acts like an excitatory input. Consequently, a short ISI will induce a stronger inhibition (adaptation) that now causes a likewise short interval and results thus in a positive correlation between adjacent ISIs. Also, the shortening effect of the adaption current in the early negative phase of the PRC can be exactly balanced by the delaying effect of the late positive phase of the PRC (pseudo-renewal case, in which the area under $\tilde{Z}$ is zero).

## 3. Discussion

We have found a general relation between two experimentally accessible characteristics: the serial interval correlations and the phase response curve of a noisy neuron with spike-triggered adaptation. The theory predicts distinct correlation patterns like short-range negative and oscillatory correlations that have been observed in experiments (Ratnam and Nelson, 2000; Nawrot et al., 2007) and in simulation studies of adapting neurons (Chacron et al., 2000; Liu and Wang, 2001).

Beyond negative and oscillatory correlations, we have found, however, that resonator neurons with spike-frequency adaptation can exhibit purely positive ISI correlations or a pseudo-renewal process with uncorrelated intervals. Adaptation currents that are commonly associated with negative ISI correlations (Wang, 1998; Chacron et al., 2001; Liu and Wang, 2001; Chacron et al., 2003; Benda et al., 2010; Nesse et al., 2010) can thus induce a rich repertoire of correlation patterns. Despite the multitude of patterns, there is a universal limit for the cumulative correlations at high firing rates [cf. Equation (11)], which shows that the long-term variability of the spike train is in this limit always reduced in agreement with experimental studies (Ratnam and Goense, 2004).

Our analytical results apply to arbitrary adaptation strength and time scale but require that (1) the noise is weak and white, (2) the deterministic dynamics shows periodic firing with equal ISIs (i.e., a limit-cycle exists) and (3) the adaptation current is purely spike-triggered with (4) a single exponential decay time. Regarding the weak-noise assumption, we found from numerical simulations quantitative agreement with our theory for values of the coefficient of variation (CV) up to 0.4, which is, for instance, typical for neurons in the sensory periphery (Ratnam and Nelson, 2000; Neiman and Russell, 2004; Vogel et al., 2005). This holds even in the subthreshold regime at low CVs, where the deterministic system does not follow a limit cycle. In this case, T^*^ has to be replaced by the mean ISI. Moreover, we found qualitative agreement even for moderately strong noise with values of the CV up to 0.8, which is typical for cortical non-bursting neurons *in vivo* (e.g. Figure 3 in Softky and Koch (1993)).

In the absence of a deterministic limit-cycle, i.e., in the fluctuation-driven regime at high CVs, different mathematical approaches have to be employed, such as those based on a hazard-function formalism (Muller et al., 2007; Nesse et al., 2010; Schwalger and Lindner, 2010; Farkhooi et al., 2011). Furthermore, for some parameter sets, we also observed repeat periods of the deterministic system that involved multiple ISIs corresponding to a periodic ISI sequence with *T*_*i*_ = *T*_*i* + *n*_, where the smallest period is *n* ≥ 2. Such cases can realize bursting (Naud et al., 2008), which we did not consider in the present study. However, we expect that these parameter regimes yield interesting correlation patterns because already in the noiseless case a periodic ISI sequence exhibits correlations between ISIs.

Regarding the last two assumptions, it seems that the analytical derivation cannot be easily extended to the cases of adaptation currents activated by the subthreshold membrane potential (“subthreshold adaptation” Ermentrout et al., 2001; Brette and Gerstner, 2005; Prescott and Sejnowski, 2008; Deemyad et al., 2012) and multiple-time-scale adaptation (Pozzorini et al., 2013). Ermentrout et al. (2001) have shown that the inclusion of subthreshold adaptation can lead to type II PRCs, which according to our theory could qualitatively change the correlation patterns. An adaptation dynamics depending on the subthreshold membrane potential also involves a fluctuating component because *v* is noisy. According to Schwalger et al. (2010), this stochasticity could contribute positive correlations. The combined effect of spike-triggered, subthreshold and stochastic adaptation currents on the sign of the SCC is not clear.

The important cases of the fluctuation-driven regime and multiple-time-scale adaptation have been recently analyzed with respect to the first-order spiking statistics including the stationary firing rate as well as the mean response to time-dependent stimuli (Richardson, 2009; Naud and Gerstner, 2012). The second-order statistics, which describes the fluctuations of the spike train (“neural variability,” cf. Section 4.2) and which limits the information transmission capabilities of neurons, is however still poorly understood theoretically in these cases. How adaptation shapes second-order statistics in the cases of multiple adaptation time scales, fluctuation-driven spiking and sub-threshold adaptation is an interesting topic for future investigations.

As an outlook we sketch, how our theory could be used to constrain unknown physiological parameters by measured SCCs and PRCs. For instance, from the mean ISI we can estimate T^*^ = 〈*T*〉. Furthermore, knowing ρ_1_ = −*A*(α, ϑ)(1 − ϑ) as well as the ratio ρ_2_/ρ_1_ = αϑ one can eliminate ϑ and solve for α. This allows to estimate the unknown adaptation time constant τ_*a*_ = −T^*^/ln α and the amplitude of the adaptation current

(17) $$a^{*}=\frac{\tau_{a}}{\alpha }\left(\alpha -\frac{\rho_{2}}{\rho_{1}}\right)/\int_{0}^{T^{*}}\text{d}t\;Z(t)e^{-\frac{t}{\tau_{a}}}.$$

Although experimental PRCs are notoriously noisy (Izhikevich, 2005), the integral over *Z*(*t*) determining our estimate of a^*^ is less error-prone. Combining our approach with advanced estimation methods for the PRC (Galán et al., 2005), may thus provide an alternative access to hidden physiological parameters using only spike time statistics.

## 4. Materials and methods

### 4.1. Phase-response curves of adapting IF models

We use the phase-response curve *Z*(*t*′) to characterize the shift of the *next* spike following a small current pulse applied at a given “phase” *t*' ∈ [0, T^*^] of an ISI. More precisely, let us assume that the last spike occurred at time *t*_0_ = 0. Then, the next spike time *t*_1_ of the perturbed limit cycle dynamics $\dot{v}=f_{0}(v,\;w)+\mu -a+\epsilon \delta (t-t^{\prime })$, *v*(0) = 0, **w**(0) = **w**_*r*_, *a*(0) = a^*^, 0 < *t*′ ≤ T^*^ will be shifted by some amount δ*T*(*t*′, ϵ) = *t*_1_ − T^*^. The infinitesimal PRC can be defined as the limit

(18) $$Z(t^{\prime })=-\underset{\epsilon \to 0}{\lim}\frac{\delta T(t^{\prime },\;\epsilon )}{\epsilon },$$

where the sign has been chosen such that a spike advance (δ*T* < 0) due to a positive stimulation (ϵ > 0) leads to a positive PRC. The definition of *Z*(*t*) by the shift of the next spike differs from the PRC that describes the asymptotic spike shift but is equivalent to the so-called “first-order PRC,” which is often measured in experiments (Netoff et al., 2012).

#### 4.1.1. Adjoint equation and boundary conditions

The PRC can be computed using the adjoint method (see e.g. Ermentrout and Terman (2010)). To this end, the dynamics is linearized about the T^*^-periodic limit cycle solution **y**_0_(*t*) = [*v*_0_(*t*), **w**_0_(*t*), *a*_0_(*t*)]. The linearized limit-cycle dynamics **y**(*t*) = **y**_0_(*t*) + δ**y**(*t*) corresponding to Equation (2) is given by

(19) $$\dot{\delta y}=A(t)\delta y$$

with the Jacobian matrix

(20) $$A(t)=\left(\begin{array}{l} \;\frac{\partial f_{0}}{\partial v}\;\frac{\partial f_{0}}{\partial w_{1}}\;\ldots \;\frac{\partial f_{0}}{\partial w_{N}}\;-1\\ \tau_{1}^{-1}\frac{\partial f_{1}}{\partial v}\;\tau_{1}^{-1}\frac{\partial f_{1}}{\partial w_{1}}\;\ldots \;\tau_{1}^{-1}\frac{\partial f_{1}}{\partial w_{N}}\;0\\ \ldots \ldots \ldots \ldots \ldots \ldots \ldots \ldots \ldots \ldots \ldots \\ \tau_{N}^{-1}\frac{\partial f_{N}}{\partial v}\;\tau_{N}^{-1}\frac{\partial f_{N}}{\partial w_{1}}\;\ldots \;\tau_{N}^{-1}\frac{\partial f_{N}}{\partial w_{N}}\;0\\ \;0\;\cdot \cdot \cdot \;0\;-\tau_{a}^{-1} \end{array}\right)$$

evaluated at *v* = *v*_0_(*t*), **w** = **w**_0_(*t*). The linear response of the ISI to perturbations of the limit-cycle dynamics in an arbitrary direction is given by the vector **Z**(*t*) = [*Z*(*t*), *Z*_*w*_1__(*t*), …, *Z*_*w*_*N*__(*t*), *Z*_*a*_(*t*)]^T^, where the first component is equal to the PRC defined above. This vector satisfies the adjoint equation **Ż** = −*A*^T^**Z** (*A*^T^ denotes the transpose of *A*) with the normalization condition ${\dot{v}}_{0}(t)Z(t)+{\dot{w}}_{0}(t)Z_{w}(t)+{\dot{a}}_{0}(t)Z_{a}(t)=1$. The remaining *N* + 1 boundary conditions are obtained by the following consideration: On the limit cycle Γ, a phase ϕ: Γ → [0, T^*^] can be introduced in the usual way by inverting the map *t* ↦ **y**_0_(*t*) and setting ϕ = t. Because we are interested in the shift of the *next* spike, it is useful to define the isochrons (sets of equal phase) as the sets of all points in phase space that will lead to the same first spike time. Put differently, phase points belonging to the same isochron will have their first threshold crossing in synchrony. As a consequence, the threshold hyperplane defined by the condition *v* = *v*_T_ is a special isochron corresponding to the phase ϕ = T^*^. Note that this definition of the phase implies that the reset line defined by the condition *v* = 0, **w** = **w**_*r*_ does generally *not* correspond to ϕ = 0 but to positive phases if *a* < a^*^ and negative phases if *a* > a^*^. Thus, off-limit-cycle trajectories suffer a phase jump upon reset. Close to the threshold, the isochrons are parallel to the threshold, and thus, a perturbation perpendicular to the *v*-direction does not change the phase. This insensitivity implies the boundary conditions *Z*_*w*_1__(T^*^) = … = *Z*_*w*_*N*__(T^*^) = *Z*_*a*_(T^*^) = 0. Note that a definition of the PRC based on the asymptotic spike shift would require periodic boundary conditions (Ladenbauer et al., 2012).

From the above considerations, it becomes clear that the PRC *Z*(*t*) can be computed for *t* ∈ [0, T^*^] by solving the system

(21) $$\left(\begin{array}{c} \dot{Z}\\ {\dot{Z}}_{w_{1}}\\ \vdots \\ {\dot{Z}}_{w_{N}} \end{array}\right)=-\left(\begin{array}{cccc} \frac{\partial f_{0}}{\partial v} & \tau_{1}^{-1}\frac{\partial f_{1}}{\partial v} & \ldots & \tau_{N}^{-1}\frac{\partial f_{N}}{\partial v}\\ \frac{\partial f_{0}}{\partial w_{1}} & \tau_{1}^{-1}\frac{\partial f_{1}}{\partial w_{1}} & \ldots & \tau_{N}^{-1}\frac{\partial f_{N}}{\partial w_{1}}\\ \vdots & \vdots & \ddots & \vdots \\ \frac{\partial f_{0}}{\partial w_{N}} & \tau_{1}^{-1}\frac{\partial f_{1}}{\partial w_{1}} & \ldots & \tau_{N}^{-1}\frac{\partial f_{N}}{\partial w_{N}} \end{array}\right)\left(\begin{array}{c} Z\\ Z_{w_{1}}\\ \vdots \\ Z_{w_{N}} \end{array}\right)$$

subject to the boundary conditions

(22) $$\;Z(T^{*})=\frac{1}{{\dot{v}}_{0}(T^{*})}=\frac{1}{f_{0}(v_{\text{T}},\;w_{0}(T^{*}))+\mu -a^{*}+\Delta },$$

(23) $$Z_{w_{k}}(T^{*})=0,\;k=1,\ldots ,N.$$

The PRC with respect to *a* is determined by

(24) $${\dot{Z}}_{a}=\frac{1}{\tau_{a}}Z_{a}+Z(t),\;Z_{a}(T^{*})=0.$$

The matrix in Equation (21) is again evaluated on the limit cycle at *v* = *v*_0_(*t*), **w** = **w**_0_(*t*) and is therefore time-dependent. An analytic solution of Equation (21) is possible for one-dimensional models with adaptation (*N* = 0) or general linear IF models although in most cases the deterministic period T^*^ still has to be computed numerically.

#### 4.1.2. One-dimensional case

In the case *N* = 0, the PRC satisfies the equation *Ż* = −*f*′(*v*_0_)*Z* with boundary condition (22). The solution is given by Equation (13). In order to prove Equation (14), we compute *Z*_*a*_(*t*) from Equation (24) yielding

$$Z_{a}(t)=e^{\frac{t}{\tau_{a}}}\left(Z_{a}(0)+\int_{0}^{t}Z(t^{\prime })e^{-\frac{t^{\prime }}{\tau_{a}}}\text{ d}t^{\prime }\right).$$

Evaluation of this expression for *t* = T^*^ leads to $\vartheta =1+\frac{a^{*}}{\tau_{a}}Z_{a}(0)$. Finally, using the normalization condition $(f(0)+\mu -a^{*})Z(0)-\frac{a^{*}}{\tau_{a}}Z_{a}(0)=1$ yields Equation (14).

### 4.2. Relation between second-order statistics of spike count, spike train and interspike intervals

A stationary sequence of spike times {…, *t*_*i* − 1_, *t*_*i*_, *t*_*i* + 1_, …} is often characterized by the statistics of the spike train *x*(*t*) = ∑_*i*_δ(*t* − *t*_*i*_), the spike count $N(t)=\int_{0}^{t}\text{d}t^{\prime }\;x(t^{\prime })$ or the sequence of ISIs {*T*_*i*_ = *t*_*i*_ − *t*_*i* − 1_}. In particular, neural variability can be quantified by the second-order statistics of these different descriptions as, for instance, the spike train power spectrum

(25) $$S(f)=\int \text{d}\tau \;e^{2\pi if\tau }\langle x(t)x(t+\tau )\rangle ,$$

the Fano factor

(26) $$F(t)=\frac{\langle N{\left(t\right)}^{2}\rangle -{\langle N(t)\rangle }^{2}}{\langle N(t)\rangle },$$

and the coefficient of variation $C_{\text{V}}=\sqrt{\langle {\left(T_{i}-\langle T_{i}\rangle \right)}^{2}\rangle }/\langle T_{i}\rangle$ and SCC ρ_*k*_ as defined in Equation (1). These statistics are connected by the fundamental relationship (Cox and Lewis, 1966) (see also (van Vreeswijk, 2010))

(27) $$\underset{t\to \infty }{\lim}F(t)=\langle T_{i}\rangle \underset{f\to 0}{\lim}S(f)=C_{\text{V}}^{2}\left(1+2\sum_{k=1}^{\infty }\rho_{k}\right).$$

It shows that the summed SCC has a strong impact on the long-term variability of the spike train. In particular, a negative sum yields a more regular spike train on long time scales than a renewal process with the same *C*_V_.

### Conflict of interest statement

The authors declare that the research was conducted in the absence of any commercial or financial relationships that could be construed as a potential conflict of interest.
